# Hypothetical Control of Fatal Quarrel Variability

**DOI:** 10.3390/e23121693

**Published:** 2021-12-17

**Authors:** Bruce J. West

**Affiliations:** 1 Center for Nonlinear Science, University of North Texas Denton, Denton, TX 76203, USA; 2 Office for Research and Innovation, North Carolina State University Rayleigh, Raleigh, NC 27695, USA; bjwest@ncsu.edu

**Keywords:** fatal quarrels, fomplexity, fractional Fokker-Planck equation, inverse power laws, Pareto, war, terrorism

## Abstract

Wars, terrorist attacks, as well as natural catastrophes typically result in a large number of casualties, whose distributions have been shown to belong to the class of Pareto’s inverse power laws (IPLs). The number of deaths resulting from terrorist attacks are herein fit by a double Pareto probability density function (PDF). We use the fractional probability calculus to frame our arguments and to parameterize a hypothetical control process to temper a Lévy process through a collective-induced potential. Thus, the PDF is shown to be a consequence of the complexity of the underlying social network. The analytic steady-state solution to the fractional Fokker-Planck equation (FFPE) is fit to a forty-year fatal quarrel (FQ) dataset.

## 1. Introduction

This essay is about distinguishing between simple and nonsimple (We avoid the use of the term *complexity* herein every we can and replace it with the term *nonsimple*. We do this for the same reasons that the term nonlinearity was introduced over a half century ago to describe dynamics that are not linear) phenomena, networks and systems, as well as why catastrophes such as fatal quarrels cannot be understood using simple models. On the one hand, characterizing some properties of social aggregates, although simple, can actually be quite complicated as distinct from nonsimple. Biological/physical properties, such as height, stamina, and strength, vary randomly from person to person and are described by normal statistics, that is, by the bell-shaped probability density function (PDF) of Gauss. On the other hand, properties such as the time intervals between successive beats of the heart, or between successive breaths, and the length of successive strides in walking typically have fluctuations with PDFs with heavy tails [1]. The latter type of size distributions are indicative of nonsimple underlying processes. Such inverse power law (IPL) PDFs were discovered and developed in the late nineteenth century by Pareto [2], who used them to understand the social imbalance revealed in the way wealth was distributed in Western society. Note that phenomena described by Gauss statistics are simple, whereas those described by Pareto statistics are nonsimple and that nonsimplicity does not uniquely restrict the mechanism other than it not be simple.

Machado et al. [3], among others, point out that such IPLs have been used to model nonsimple phenomena whose underlying dynamics are fractal, have long-term memory and are often described by the fractional calculus. An extensive review of IPL phenomena and how they are modeled using the fractional calculus is given by West and Grigolini [4]. Subsequently, the way one solves real-world nonsimple problems is determined by how one thinks, using the mental tools constructed from our experience of the Gauss and Pareto types of PDFs.

**Figure 1:**
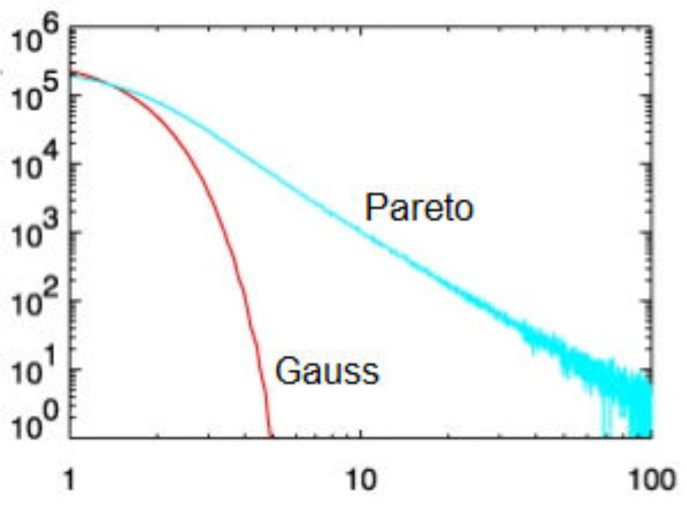
The single humped PDF of Gauss is compared with the IPL PDF of Pareto on log–log graph paper. The log of the relative frequency emphasizes the exponential decrease on either side of the average value in the Gauss PDF compared with the IPL tail of the Pareto PDF.

Figure 1 schematically compares the bell-shaped distributon of Gauss and the IPL distribution of Pareto, obtained from appropriately chosen random walks. The horizontal axis is the distance traveled by a random walker in arbitrary units and the vertical axes is the logarithm of the number of trajectories (histogram) at that distance. The PDFs differ by orders of magnitude for equal distances traveled by the two kinds of walkers. Consider the situation where the distance x=3 represents an extreme, say a failure threshold for the underlying process. The failure is more than an order of magnitude more likely to occur for a process described by Pareto than by Gauss statistics. If the process is considered to be simple, then the preparations one makes for the occurrence of a catastrophic extreme event would be woefully inadequate if it was in fact nonsimple. Things only get worse for x>3.

We emphasize that the nonsimple problems facing society can only be solved by adopting a way of thinking that goes beyond simple logic and recognizes the fundamental nonsimplicity of collections of individuals. Moreover, the simple *either/or* solutions of the past have often led to false dichotomies, such as ‘boom or bust’, ‘feast or famine’ and ‘war or peace’. These descriptive dichotomies of outcome reflect the confined range of thought used to solve the problem with which decision makers were confronted. As a consequence of the kinds of action leaders are willing to consider the choice made is often disastrous because policy makers are either unwilling or unable to think outside a box of restricted options. Catastrophes can often be avoided by exercising a counter-intuitive third option of a *both/and* choice [5] outside the confines of the *either/or* options. Thus, nonsimple logic of paradox thinking is a strategy that enables policy makers to anticipate the next social contradiction and to resolve a paradox in this arena before it materializes.

In Section 2, the IPL of Pareto is introduced and its interpretation in terms of a social imbalance is presented. The implications of Pareto’s Law are explored in the context of the uncertainty generated by the social instability of a nation going to war and how war is entailed by the false either/or choice in the way we think about solving “wicked problems” (Wicked Problems have a number of charateriestics as listed on Wikipedia [6]: (1) The problem is not understood until after the formulation of a solution. (2) Wicked problems have no stopping rule. (3) Solutions to wicked problems are not right or wrong. (4) Every wicked problem is essentially novel and unique. (5) Every solution to a wicked problem is a “one shot operation”. (6) Wicked problems have no given alternative solutions.). The cognitive maps we develop based on simple and nonsimple statistics are developed further and the replacement of the *either/or* choice by the counter-intuitive *both/and* choice is discussed. In Section 3, the hypothesis of control of the emergent behavior of nonsimple network model of terrorist attacks on sovereign nations is introduced. Richardson’s fatal quarrel variability (FQV) dataset is fit by the steady-state solution to the fractional Fokker–Planck equation given by a two-slope IPL PDF for the number of deaths due to terrorists attacks over a four decade period ending in 2009. A number of conclusions are drawn in Section 4.

## 2. Pareto’s Law

Datasets on the distribution of wealth [7], the richness of language [8], the robustness of urban growth [9], the number of deaths due to fatal quarrels [10], the intrinsic flexibility of biological macroevolution [11] and the inherent variability of other nonsimple phenomena are not statistically normal, which is to say that their statistics are not given by the PDF of Gauss and they are not simple. Instead, they are described by IPL PDFs with long tails and consequently entail an imbalance in the underlying process. Historically, this imbalance in the distribution of wealth, for example, was depicted by arbitrarily assuming that a small fraction of the population, say 20% (The 20% value caught-on and is used to represent processes whose imbalance can be as small as 1%. So remember it is the existence of the IPL index that creates the imbalance not its magnitude.), owns a large fraction of the stuff worth having, say 80% of social wealth. This argument was generalized over the twentieth century from Pareto’s first articulation of the empirical distribution of wealth in the late nineteenth century and became known as the 80/20 Principle [12]. Subsequently, Pareto’s IPL PDF has been effectively applied to all manner of nonsimple phenomena [4].

The actual numerical value of the IPL index which is responsible for the specific partitioning of a particular dataset is not necessarily important, but the existence of the Pareto imbalance resulting from the datasets that determine these index values is crucial in interpreting the behavior of the underlying processes. The larger the Pareto index, the steeper the slope of the IPL and the smaller the imbalance. The smaller the index, the shallower the slope of the IPL and the greater the imbalance. The imbalance and the nonsimplicity of the process move in direct proportion to one another and inversely with the IPL index. This imbalance in the nonsimple networks has led to the complexity matching effect (CME), where the interaction of two nonsimple networks produces a flow of information from the more to the less nonsimple network [13,14]. This has led to new ways to facilitate post-operative rehabilitation by means of replacing harmonic mechanical ventilation with biologically variable ventilation [15], as well as other kinds of rehabilitation [14], including the mitigation of walking pathologies resulting from neurodenerative diseases [16].

The 80/20 Principle is also known as Pareto’s Law and a person seeking success in today’s society needs to learn how to leverage this imbalance in the appropriate social variables to their advantage. In large organizations, the imbalance expressed by Pareto’s Law is often manifest in the distribution of achievement translating into most of the individual accomplishments being generated by a minority of the organization’s members. The “vital few” have the talent and ability necessary to generate innovative ideas and carry them to fruition. These individuals provide the leadership to focus the attention and activity of the majority on the tasks required to produce results, whether these results are new ways to carry out old tasks, or they are new tasks resulting in totally different mission strategies, both requiring new ways of thinking.

In any large group, it is the 20% that produce most of the results, but it is not always the same 20%. There is a flux from the many to the few and back again, as recognized by Pareto, and only the rarest of individuals remains permanently within the ranks of the few. Consequently, a successful CEO or military policy maker values the support role of the 80%, as well as being prepared to step into or out of the 20% role at a moment’s notice, as the need arises.

As a large organization, the military is no different from any other large organization in its distribution of talent, achievers, trouble makers and problem solvers. The nonsimple problems inhibiting the success of any organization, or of a mission within the military, often appear to be independent of one another, but in point of fact, they are not. Nonsimple problems, are interwoven, however subtly, so that generating solutions is closely related to generating results to apparently unrelated problems. What this means in practice is that the most valuable solutions are connected to multiple problems within an organization; the distribution of solutions have the same imbalance in their influence on problems, as individual talents have on the distribution of income among people. This imbalance is manifest in 80% of the problems being resolved by 20% of the solutions. These are the valuable solutions the successful CEO and policy maker identify, generate and exploit; find the solution that not only solves the problem of immediate concern, but also facilitates the solution of problems that, although not immediately evident, can be anticipated.

The linear properties of proportionality and additivity are lost in essentially all nonsimple systems, as observed by von Clausewitz [17] regarding war:

*The scale of a victory does not increase simply at a rate commensurate with the increase in size of the defeated armies, but progressively. The outcome of a major battle has a greater psychological effects on the loser than the winner. This, in turn, gives rise to additional loss of material strength [through abandonment of weapons in a retreat or desertions from the army], which is echoed in loss of morale; the other two become mutually interactive as each enhances and intensifies the other.*

Natural phenomena are typically nonlinear, which implies that their behavior is intrinsically uncertain with unexpected changes, such as in the asymmetric response to losses in battle observed by von Clausewitz. His observation of the asymmetric response to victory is another manifestation of Pareto’s Law. This way of thinking avoids undo expectation, surprise and disappointment, which are three precursors to failure. Expectations are typically formed by linearly extrapolating from the present situation into the unknown future and therein lies the vulnerability. The occurrence of an unexpected event is the source of surprise, which if sufficiently bizarre may inhibit a linear response and instead provoke a disproportionate one, resulting in a catastrophically negative outcome. Alternatively, the response may be upset and disappointment, resulting in a clouding of the needed situational awareness, setting the stage for a future catastrophe.

The new perspective on thinking emphasizes the need to understand how nonsimple networks interact with one another, exchange information and adapt. The key idea here is to adapt with increasing situational awareness rather than merely reacting in the face of changing adversarial behavior. In the military the inability to understand nonsimplicity is summarized in the phrase `fog of war’, indicating the overwhelming nonsimplicity of the battlefield and the lack of ability of any single individual to truly comprehend that nonsimplicity as it develops over time. Perhaps a new way of thinking will enable the military policy maker and war fighter to see further than previously, if not to lift the veil of fog all together.

**Figure 2:**
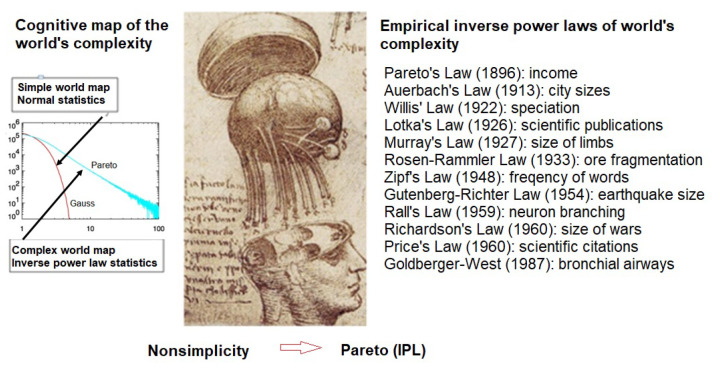
Here we use an image from da Vinci’s anatomical studies to emphasize the brain’s mapping of the experienced world. On the left, the world map of normal statistics is contrasted with that of Pareto’s IPL statistics. On the right are a dozen empirical IPLs along with the names of the scientists that did the original analysis. In each case, it was determined that the nonsimplicity of the underlying phenomenon implied the Pareto IPL PDF. Some of the references useful in the present context are Lotka [18]: number of scientists publishing a given number of papers; Zipf [8]: the rank ordering of word frequency in a language; Price [19]: fraction of scientific papers with a given number of citations published in a year; Auerbach [9]: number of urban population centers of a given size within the United States at its origin; Willis [11]: number of species of flower within a given taxa; Pareto [2]: distribution of income in Western countries at the turn of the twentieth century.

People’s experiences of events are condensed and recorded as part of cognitive maps along with a narrative of how these individual map elements are related to one another. Regardless of the representation used, the ordering of events and perhaps even their relative separation in space and time is mostly retained; see Figure 2. Thus, the feeling of satisfaction, or excitement, experienced by a person may be a consequence of the resonance between a pattern in a person’s mental map and the experienced ordering within the dataset in the outside world. That resonance may be captured in an author’s use of language and the frequency with which certain familiar words are used, such that the tale being told flows at an uninterrupted pace and it is a pleasure to lose oneself in the story. This CME might also reflect the experience of `being in the groove’, where an individual is able to carry out a sequence of nonsimple tasks in effortless fashion that at other times requires a great deal of effort [13].

A war fighter on a battlefield is faced with the prospect of spending an ever-decreasing store of resources to protect against an ever-increasing likelihood of catastrophe and must make decisions by balancing what is in inventory, against what is reasonable to lose fighting the adversary. The immediate difficulty is determining how to balance the likelihood of a catastrophic event against the cost of mitigating against it. The only way to rationally construct such a trade-off, between the balance of cost against peace of mind, is to have a systematic estimate of the probability for the occurrence of an extreme event, an estimate that may never have been measured. If, like most people in society, the warfighter believes that catastrophes are rare events and therefore unlikely, this is followed by thinking that extremes are few and far between. Such conclusions are built up from everyday experience of stubbing one’s toe rather than the extreme of breaking a leg, of having a disagreement with one’s boss and not being fired; in short, catastrophes are few and far between in the lifetime of most people in a stable society.

However, when a nation goes to war, its society becomes unstable, and the experience of the likelihood of catastrophe for the typical individual increase dramatically. The small, relatively harmless, changes from the normal routine is a thing of the past and any deviation brings on anxiety focusing on the expectation of calamity. In this unstable social situation the normal statistics of Gauss are replaced with the IPL statistics of Pareto. Very often, such heavy-tailed distributions are a consequence of intermittence in the underlying dynamics, so that large swings in the observable occur more often than would be expected from the stable dynamics of simple systems, such as depicted in Figure 2. So, what does an engineer do when the statistics of the underlying process are not bell shaped? Or what does a typical warfighter do for that matter?

Simple phenomena, with Gauss statistics, have extrema that are relatively easy to prepare for, because they are rare. Nonsimple phenomena, with IPL statistics, produce extrema of a given large size much more frequently than do simple statistical processes and require special consideration along with major investments of resources in order to prepare for their almost certain occurrence.

Unfortunately, when nonsimple phenomena are mistakenly described using a Gauss PDF, society is ill prepared for the real-world extreme events when they occur. This is what happened repeatedly with the stock market crashes of the last century and early in this century; each time a market crash occurred, it was a surprise, and subsequently the crash was treated as an anomaly for which no one was to blame. Such events ought to have been anticipated, but they never were, even though their existence is known to be an intrinsic part of stock market dynamics [20]. This shortsightedness and inadequate preparation must be avoided on the part of the warfighter and it can be, by using paradox thinking. For example, a warfighter anticipating when the next attack will occur is like waiting for the next drop of water from a leaky faucet to come crashing down, since the time intervals between the release of consecutive water drops is an IPL PDF [21]. The occurrence of either kind of event has a distribution with a long tail; the cost of survival is eternal vigilance, increased sensitivity, and a touch of paranoia.

Thus, the datasets on the distribution of wealth, the frequency of word use in language, city sizes, biological macroevolution and apparently most other nonsimple phenomena have distributions that are more like the IPL of Pareto than they are like the Gauss bell-shaped curve of error analysis. The existence of the upward mobility on the part of individuals that is observed in social development, as opposed to biological development, is a consequence of the nature of the intrinsic constraints on the underlying process. The ceiling is hard-wired in biology, such as the maximum physical height attainable by an individual, but the constraints are much more flexible in sociology, where a person of any height can acquire great wealth. This difference argues for the adaptive response of the individual in an extremely hostile social situation being potentially victorious, a situation that Taleb named *antifragility* [20]. This is the opposite of being fragile. Antifragility is the ability to gain strength from the chaos of the environment which is not the same as resilience to chaos since one can do more than just survive, one can actually grow stronger from these hostile interactions with the environment.

Note that we have explicitly identified Pareto’s imbalance, resulting from the IPL, in the spread of wealth and other nonsimple phenomena, as depicted in Figure 2. The former appears to be determined by the nonsimplicty of society, rather than by a natural constraint, and that suggests we should be able to modify the PDF, or at least modify the reaction of key individuals to the surprise occurrence of an event. This has been done with varying degrees of success through the implementation of all manner of social regulation, e.g., to redistribute wealth more equitably; the graduated income tax comes to mind. However, a minimal imbalance seems necessary, since it resists all efforts at equalization through its removal; either by equalizing all income levels, or putting virtually 100% of the wealth in the hands of the few, which appears to destabilize society entirely [7]. Thus, it ought to be possible to modulate the outcome of FQs without changing its fundamental behavior.

## 3. Wars, Deaths and IPLs

It has been known since Richardson first published his examination of the distribution of FQs [22] that the number of deaths *x* in wars, insurgencies, as well as in acts of terrorism, all of which are included in his definition of a FQs, follow approximate IPL PDFs:

(1) $$P\left(x\right)x^{-\alpha }.$$

We refer to the *x*-process as fatal quarrel variability (FQV). The FQV PDF is defined for $x_{\min}\leq x\leq \infty ,$ where $x_{\min}$ is estimated along with the IPL index α from existing datasets [23]. Consequently, the intuition developed for Pareto’s Law can be used to interpret the statistical behavior of distinct groups within conflicts. For example, Bohorquez et al. [24] established a discrete spectrum of IPL indices whose specific values depend on the nature of the conflict. They determined that conventional wars have an IPL index with the value α=1.8 and that global terrorism has the IPL index α=2.5. Their theory of fragmentation and coalescence processes of attack units is consistent with the critical dynamics that occurs in nonsimple networks and enabled them to interpret the smaller IPL index as being the result of a tendency toward building larger more robust attack units with fixed attack strength. A characteristic of conventional war. The larger IPL index results from on-going fragmentation and coalescence process resulting in transient attack units for terror against G7 targets. Therefore unlike a conventional army, a terrorist group is more likely to form smaller attack units than larger ones. The study of nonsimple networks suggests that the nonsimplicity of a phenomenon increases with decreasing IPL index in agreement with the long standing observation that terrorism is simpler than conventional war.

**Figure 3:**
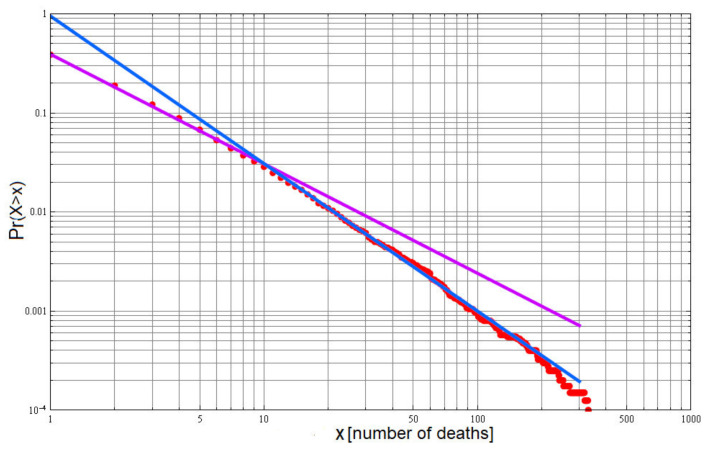
The FQV probability is IPL, $P\left(X\geq x\right)\propto 1/x^{\alpha -1}$. The severity of an event is greater than a given amount is depicted using data on the FQ deaths due to terrorist attacks between 1968 and 2009 [25]. When graphed on log–log graph paper, the FQV probability yields a straight line with negative slope α−1. The FQV dataset produces a double Pareto PDF [27] with IPL indices of α=2.11 and 2.49 :  $P\left(X\geq x\right)=\frac{0.39}{x^{1.11}}U\left(10-x\right)+\frac{0.95}{x^{1.49}}U\left(x-10\right)$, where U(z)=1 for z>0 and = 0 for z<0.

For the present discussion, we display the global FQV in terrorist attacks, between 1968 and 2009. There were in excess of $2\times 10^{4}$ terrorist attacks world-wide and the number of dead, or injured, were determined to follow IPL distributions [25]. Depicted in Figure 3 is the logarithm of the number of those killed given on the horizontal axis and the vertical axis is the logarithm of the probability that this number exceeds the number on the horizontal axis. It is noted that rare catastrophic attacks are not outliers of an otherwise simple process, nor are they exceptions to the rule: they are the rule [26].

### FQV Control Hypothesis

The FQV time series recording the number of deaths during a fatal quarrel is determined by a number of control mechanism that shape the conflict. These controls are a consequence of the strategies implemented by each side to minimize their own casualties and maximize those of the opposition. A hypothetical dynamic equation for the FQV time series is formally expressed by the nonlinear Langevin equation (NLE):

(2) $$dX\left(t\right)=F\left(X,t\right)dt+d\xi \left(t\right),$$

where F(X,t) is a deterministic driver and dξ(t) is a stochastic driver. Both these drivers can be made functionally specific by using the known properties of FQV datasets.

In the physics literature, it is customary to distinguish between the phase-space variable *x* and the dynamic variable X(t) or $X(t,x_{0})$ describing the solution trajectory at time *t* initiated at $x_{0}=X\left(0\right)$. The phase-space function for the trajectory is defined $\rho \left(x,t\right)\equiv \delta \left(x-X(t,x_{0}\right)).$ The deterministic driver incorporates the dynamics determined by military policy, training and discipline, which determine the interactions among individuals in a nonsimple social network. The random driver consists of the erratic fluctuations in the adversary’s number due to deaths, injuries and desertions. The solution to the NLE is consequently a random variable with the corresponding probability density function (PDF):

(3) $$P\left(x,t\right)={\langle \delta \left(x-X(t\right))\rangle }_{\xi },$$

where the subscripted brackets denote an ensemble average over the stochastic driver and the dependence on the initial condition is suppressed.

The FQV time series results from the competing aggressiveness on the part of the members of the conflicts. The competition is assumed here to provide the erratic variability recorded in the number of fatalities in each of the FQs and therefore determines the statistics of the stochastic driver. Thus, we assume the statistics of the stochastic driver in the NLE dξ(t) resulting from the competition between the IPL FQ inputs to be one-sided Lévy stable (OSLS).

The form of the deterministic driver F(X,t) in the NLE emerges from the intrinsic dynamic properties of the members of the collectives engaged in the conflict. These properties are modeled herein using the recent observation that nonsimple social networks are poised at criticality [13], which can in principle produce cooperative oscillations, as well as erratic variability, observed in the behavior of time series in stable social situations. However, the fluctuations arising from the chaotic microdynamics are assumed to be overwhelmed by the OSLS statistics of the random driver and therefore are ignored in the NLE. The deterministic force is therefore modeled by the cooperative behavior of the belligerent parties that keeps the FQV from exploding into a world-wide conflagration.

In physical systems, the fluctuations and linear dissipation have a common origin and consequently are tied together by a fluctuation-dissipation relation. In sociological systems, however, the dissipation and the random force often have different origins and can therefore be independent. Moreover, for the social network to remain stable, the feedback regulates the influence of the dynamic response to the random driver. In the case of FQV the deterministic force is here assumed to be given by the collective behavior of those involved in the conflict and to emerge from but not be reductionistically related to the conflict of individuals. Such cooperative behavior has been shown to be generic for large classes of nonsimple networks including members of the Ising universality class [28]. One such model near criticality is adopted below to describe the control of the dynamics of the aggregates of FQs [25].

A generic version of Equation (2) was analyzed by Chechkin et al. [29] in which the deterministic term was given by the nonlinear force F(X,t)=$-\lambda \left(X\right)X$:

(4) $$dX\left(t\right)=-\lambda \left(X\right)Xdt+d\xi \left(t\right),$$

where the fluctuations driving the system dξ(t) have OSLS statistics and the dissipation parameter λ(X) is state dependent. West and Turalska [30] subsequently recast the argument given in [29] to model the control of heart rate variability in order to understand certain kinds of cardiac pathologies. We adapt the arguments presented in [30] to the FQV’s dataset and choose the statistics of the random driver to be OSLS because we know that a linear Langevin equation (LLE) with such a random force yields an IPL PDF [31]. Equation (4) reduces to a LLE with $\lambda \left(X\right)\to \lambda_{0}=$ *constant* >0 as X→0. In general the feedback term is a polynomial, but we restrict our analysis to the two lowest-order terms, since these terms dominate their respective asymptotic regimes the control process and write:

(5) $$\lambda \left(x\right)\;=\;\lambda_{0}+\lambda_{\nu }x^{\nu -\beta },$$

where $\lambda_{\nu }>0$ and taking into account the OSLS IPL index β we have ν>β which is determined from the FQV dataset.

The initial slope of the FQV PDF is determined by the Lévy index of the random force in the nonlinear LE (NLE) which is stabilized by the parameter $\lambda_{0}.$ The second slope is determined by the nonlinear term in the control process. We consider the NLE given by Equations (4) and (5) to be generic in that the random driving force is a OSLS process resulting from the generalized central limit theorem applied to an IPL statistical process, that being the random number of FQ deaths driving the rate equation. This argument enables us to use the fractional probability calculus to replace the NLE with the fractional Fokker–Planck equation (FFPE) for the FQV PDF [5,14]:

(6) $$\frac{\partial P(x,t)}{\partial t}=\frac{\partial }{\partial x}\left[\lambda \left(x\right)xP\left(x,t\right)\right]+D\partial_{\left|x\right|}^{\beta }\left[P(x,t)\right],$$

where $\partial_{\left|x\right|}^{\beta }\left[\cdot \right]$ is the Riesz–Feller fractional derivative, 0<β<2, which can be expressed in the equivalent form [29]:

(7) $$\partial_{\left|x\right|}^{\beta }\left[P_{ss}\left(x\right)\right]=-C_{\beta }\frac{d^{2}}{dx^{2}}\int_{\Omega }\frac{P_{ss}\left(x^{\prime }\right)dx^{\prime }}{{\left(x-x^{\prime }\right)}^{\beta -1}},$$

with the coefficient $C_{\beta }=\frac{1}{2\Gamma \left(2-\beta \right)\cos\left(\pi \beta /2\right)},$ and Ω is the range of the random variable. Of course, we cannot solve Equation (6) in general, but it is not necessary to have the exact solution in hand to obtain the information we need; the time-asymptotic behavior of the steady-state solution is sufficient. Consider the steady-state FQV PDF:

(8) $$P_{ss}\left(x\right)=\lim_{t\to \infty }P\left(x,t\right)$$

given by the solution to steady-state FFPE with zero-flux boundary conditions:

(9) $$\lambda \left(x\right)xP_{ss}\left(x\right)-DC_{\beta }\frac{d}{dx}\int_{\Omega }\frac{P_{ss}\left(x^{\prime }\right)dx^{\prime }}{{\left(x-x^{\prime }\right)}^{\beta -1}}=0.$$

We assume that the solution to Equation (9) consists of two asymptotic pieces joined at some intermediate value of *x*.

Chechkin et al. [29] argue that the steady-state PDF can be determined from an analysis of the contributions to the integral in Equation (9) in the vicinity of the pole. In particular, the dominant contribution to the integral arises from the region to the left of the pole $x=x^{\prime }$ with the highest power of *x* in the dissipation function and consequently Equation (9) reduces to the large-scale asymptotic form:

(10) $$\lambda_{\nu }x^{\nu -\beta +1}P_{ss}\left(x\right)-DC_{\beta }\frac{d}{dx}\underset{1}{\int^{x}}\frac{P_{ss}\left(x^{\prime }\right)dx^{\prime }}{{\left(x-x^{\prime }\right)}^{\beta -1}}=0,$$

whose solution is given by:

(11) $$\lim_{x\to \infty }P_{ss}\left(x\right)∼\frac{DC_{\beta }}{\lambda_{\nu }x^{\nu +1}},$$

in agreement with the solution obtained by Chechkin et al. [29].

Note that this piece of the total solution must be connected with that at the small-scale asymptote x→1 where the LLE dominates and yields:

(12) $$\lim_{x\to 1}P_{ss}\left(x\right)∼\frac{DC_{\alpha }}{\lambda_{0}x^{\beta +1}}.$$

The approximate steady-state solution to Equation (9) is therefore given by the solution to Equation (9), with the upper limit on the integral replaced by x, to be the double IPL PDF [29,30]:

(13) $$xP_{ss}\left(x\right)=\left\{\begin{array}{c} \frac{N_{<}}{x^{\beta }}\;\;\;;\;1\leq x\leq 10\;\\ \frac{N_{>}}{x^{\nu }}\;\;\;\;\;\;;\;\;\;\;\;\;x\geq 10 \end{array}\right.\;\;,$$

where $N_{<}$ and $N_{>}$ are the normalization constants over the indicated *x* intervals and ν is chosen such that the steady-state distribution is continuous at the change in slope point x=10.

Chechkin et al. present a plot of $ln[xP_{ss}\left(x\right)]$ versus ln[x] and obtain a two slope graph identical to that given in Figure 3; in this way, the IPL index in $xP_{ss}\left(x\right)$ is the same as in Pr(X≥x) with the initial slope value β and the asymptotic slope given by ν>β. Applying their reasoning to the empirical dataset depicted in Figure 3, the empirical Lévy index is β≈1.11 and the asymptotic slope is ν ≈ 1.49.

## 4. Discussion and Conclusions

It is clear from Figure 3 that the terrorism FQV dataset is well fitted by two distinct IPLs making up the FQV PDF. The asymptotic FQV PDF has a slope in agreement with the studies involving terrorism where non-G7 countries are targets α=2.5(≈ν+1), and the FQV IPL is in agreement with the value obtained for North America by Becerra et al. [32]. It is of interest to note that when the double Pareto PDF is generated by a single multiplicative Langevin equation that the two IPL indices are related to one another as the two roots of a quadratic equation [27]. However, when the source of the two slopes is a consequence of a balance between a deterministic and stochastic force, the balance determines any relation between the IPL indices.

Returning to the earlier theme of this essay, the emergence and resolution of paradox can be explained qualitatively, using the difference of the properties between the bell-shaped Gauss and long-tailed Pareto distributions. Recognizing the dramatic differences in the two types of distributions suggests that paradox results from the misinterpretation that the datasets generated by nonsimple phenomena can be interpreted as if they were simple. The failure to take into account the full range of variability contained in the process is what leads one into paradox and is a consequence of using simple linear thinking to interpret a dataset that ought to be interpreted using nonsimple paradox thinking.

The distinction made between simple logical thinking and nonsimple paradox thinking at the level of a military policy maker within a bureaucracy, is equally valid at the level of the individual warfighter on the battlefield. It is nonsimple thinking that resolves paradox. It is useful to have in mind various ways paradox comes about in the military. Some representative paradoxes can be lifted directly from The *U.S. Army/Marine Corps Counterinsurgency Field Manual* and are presented here as examples of the different mindsets required for their resolution. These COIN paradoxes are offered to stimulate, not to limit, thinking and are discussed in detail in [33]:

- Sometimes, the more you protect your force, the less secure you may be.
- Sometimes, the more force is used, the less effective it is.
- Sometimes doing nothing is the best reaction.
- Some of the best weapons for counterinsurgents do not shoot.
- The host nation doing something tolerably is normally better than us doing it well.

Within a bureaucracy, the role of leadership is to support opposing internal forces and harness the continuous tension between them, enabling the system to not only survive, but to continuously improve. Of course this is the ideal case, and all too often bureaucracies become bogged down at the level of survival and need to be jolted out of their complacency. The proper management of the tensions of paradox, whether within individuals, groups or organizations produces flexibility and resilience, while fostering more dynamic decision making, without becoming doctrinaire. This replacement of Aristotelian logic entails new Principles of War, for example, the existence of a Gray Zone [21] to mitigate the *either/or* choice between war and peace and accept *both/and* as the resolution of the false dichotomy.

A leader establishes a dynamic stability within the organization that embraces paradox, one that persists over time. There always exists a tension between the short-term needs of the warfighter, particularly on the battlefield, and the long-term needs of the military to win the conflict and prepare for the future. This is the delicate balance between stability and change in the military. The seductive attraction of consistency in decision making, mistakenly taken for stability, must be avoided. A leader recognizes the desirability of inconsistency and holds multiple, conflicting views in order to spontaneously transition from *either/or-thinking* to *both/and-thinking*, to satisfy competing demands of the long term.

Of course, this NLE model would be of little interest to us here if it was dependent on the details of the interactions within the nonsimple social networks describing conflict. The dynamic variable is the aggregate of all deaths due to the separate terrorist attacks from 1968 to 2009 [25] across the globe and does not represent the microdynamics of the individual conflicts. Rather, it is assumed that the NLE describes the macrodynamic behavior that emerges from the critical dynamics of the conflict between the nonsimple social networks. Nonsimple networks that undergo phase transitions manifest scaling, which results from the universality of the underlying microdynamics. Consequently, the form of the dynamic equations in the vicinity of the critical point is also universal; see Landau’s discussion of critical slowing down when a fluid transitions from laminar to turbulent flow [34]. This is the kind of control processes hypothesized here, one that controls the emergent dynamic behavior at the macro level of two or more nonsimple networks in conflict.

We close these remarks with the observation that there is a significant difference between a leader who analyzes disputes like a chess master and one who approaches conflict like a poker player. In both, there are well-defined rules of engagement, as well as strategies for gaining position, making sacrifices, and attacking. However, in poker, there is the added dimension of deceit. Since not all the play is visible to everyone, there is the opportunity to bluff and exploit your opponent’s psychological reaction to uncertainty. In the real world, a “consistently inconsistent” devious player has an advantage over a predictable rule follower. This is where nonsimple paradox thinking, or a paradoxical frame of mind, can overcome an adversary hampered by the belief that cognitive dissonance is to be avoided [33].

## Data Availability

The data used is open source and available at [25,26].
